# Post‐COVID Hypertension With Hyperreninemic Hyperaldosteronism in a Pediatric Patient

**DOI:** 10.1002/ccr3.70119

**Published:** 2025-01-14

**Authors:** Vivian Shi, Teresa Trinka, Mohammed Faizan

**Affiliations:** ^1^ Department of Pediatrics Hasbro Children's Hospital Providence Rhode Island USA; ^2^ Warren Alpert Medical School of Brown University Providence Rhode Island USA

**Keywords:** COVID, hypertension, pediatrics, RAAS

## Abstract

COVID‐19 infection may predispose patients to long‐term renovascular complications. Early recognition and management are crucial for optimal outcomes. This case report underscores the importance of thorough follow‐up care for children recovering from COVID‐19, with a focus on monitoring blood pressure and renal function to mitigate long‐term renovascular disease.

## 1 Introduction

The coronavirus disease 2019 (COVID‐19) pandemic has affected millions of individuals globally, with disproportionately higher risk of morbidity and mortality in adult populations. Children and young adults seem to be at lower risk for severe infection; however, there has been increasing awareness of potential long‐term disease complications such as MIS‐C, in addition to neurologic, cardiac, and respiratory problems. We present a case of mild COVID‐19 infection complicated by persistent hypertension.

## 2 Case History/Examination

The patient is a 12‐year‐old overweight but otherwise healthy boy, referred to pediatric nephrology for evaluation of a 10‐month history of persistent hypertension. The patient was previously healthy until a symptomatic COVID‐19 infection in August 2020. His viral symptoms included 1 week of lethargy, headache, sore throat, and high fevers. These were symptomatically managed at home. Within 1 month following infection, he was seen for a routine visit with his primary care physician, with notable new hypertension (150/80, 140/60) and a recommendation for lifestyle modifications, including dietary changes and weight loss. Over the next 4 weeks, he achieved a 7lb. weight loss. However, on repeat evaluations in the office, he continued to have elevated blood pressures with a significant blood pressure differential between the right and left brachial arteries (162/76 and 144/76, respectively). The patient is cared for by his maternal grandmother, who reported that he had been on a healthy diet and exercising more frequently. He had no prior history of hypertension. He was referred to pediatric cardiology in October 2020 for evaluation. His evaluation included a transthoracic echocardiogram, with a wide, open‐appearing aortic arch, no significant flow acceleration, and a normal flow pattern. He had normal LV size and shortening fraction (43%). There was no evidence of left ventricular hypertrophy or aortic coarctation. He had a normal EKG. He was subsequently referred to Pediatric Nephrology for further evaluation.

Birth history is notable for premature delivery at 34 2/7 weeks. Birth weight was average for gestational age. He was admitted to the neonatal intensive care unit (NICU) for his gestational age with a 13‐day stay requiring a gavage tube for feeding/growing. He otherwise has no formal medical history. Immunizations are up to date. He takes no daily medications. Family history is notable for maternal grandmother with presumed essential hypertension, mother with depression and bipolar disorder, otherwise no congenital heart disease, sudden unexplained death, early coronary artery disease, or renal disease. The patient is in the 7th grade. He engages in a form of exercise daily, such as walking or bicycle riding.

## 3 Methods

On further investigation by Pediatric Nephrology, ambulatory blood pressure monitoring results revealed stage 1–2 range hypertension with average BP 136/71 (range 113–168/52–107), daytime average 135/70, and nighttime average 140/73. Plasma renin activity was elevated at 4.2 ng/mL/h (reference range: 0.5–3.3), aldosterone 37.6 ng/dL (reference range: 4.0–31.0). Protein creatinine ratio (0.12), Urinalysis (UA), Thyroid Stimulating Hormone (TSH) (1.761), Basic Metabolic Panel (BMP) (Na 139, K 4.0, Cl 104, CO_2_ 23, AG 12, BUN 8, Cr 0.50) and Complete Blood Count (CBC) were all within normal limits. Plasma metanephrines 0.22 (0.00–0.49 nmol/L) and normetanephrine 0.36 (0.00–0.89 nmol/L). Renal ultrasound with AV Dopplers showed right kidney at 11.1 cm in length, left kidney at 11.8 cm in length without solid mass, calculus or hydronephrosis. Intrarenal arterial/venous as well as renal arterial/venous waveforms were within normal limits and symmetric bilaterally. Abdominal ultrasound with incidental hepatic steatosis. Renal Magnetic Resonance Angiography (MRA) showed widely patent, single renal arteries bilaterally with no evidence of hemodynamically significant stenosis.

## 4 Conclusion and Results

On follow‐up visit with nephrology, the patient's weight was noted to be 76.8 kg (169 lbs.) at 98th percentile for age, BMI 31.04 at 99th percentile for age, with an overall 20‐lb. weight gain in 8 months. In the setting of elevated renin/aldosterone, the patient was started on lisinopril 5 mg once daily. He has been taking 5 mg of lisinopril as prescribed without any reported side effects and with normal‐range blood pressures. He is continuing to implement lifestyle changes such as the DASH diet and an increased level of physical activity.

## 5 Discussion

With recent research advances, current classifications of the renin‐angiotensin‐aldosterone system (RAAS) consists of a two‐arm, counter‐regulatory system divided into the classical and alternative axes. The liver is the primary organ for synthesis of angiotensinogen (AGT) which serves as the substrate of renin. The juxtaglomerular apparatus of the kidney produces renin enzyme into circulation in response to various stimuli. In the bloodstream, AGT is cleaved into multiple vasoactive peptides that serve to regulate sodium/potassium balance, body fluid volume balance as well as arterial blood pressure homeostasis. In the classical system, downstream effects of ACE/AngII/AT1R are increased aldosterone synthesis and systemic hypertension as opposed to the alternative counter‐regulatory system, where downstream effects of ACE2/Ang‐ 1–7/MasR are decreased aldosterone secretion and blood pressure. The RAAS system also influences cellular proliferation and inflammatory cascades [1]. Existing data support the prominent role of classical RAAS axis activation in the pathophysiology of many systemic illnesses and kidney damage. Since the outbreak of SARS‐CoV2, there have been several studies postulating the function of RAAS imbalance in the disease's clinical course. It is now known that viral entry is dependent on binding of the viral spike glycoprotein S to membrane‐bound ACE2, leading to the entry of ACE2 into the cell, thereby reducing the amount of active enzyme of the alternative, anti‐inflammatory RAAS axis [2]. In pediatrics, the number of confirmed COVID‐19 cases is low; approximately 17.9% of all cases occurred in patients less than 18 years of age within the United States [3]. There is also evidence for age‐dependent expression of ACE2 in the nasal epithelium, with progressively higher levels in adulthood than in children, which may also correlate with lower infectivity of COVID‐19 in younger patients [4]. Indeed, the upregulation of the classic axis is hypothesized to influence many of the clinical manifestations of COVID‐19 including stimulation of pro‐fibrotic, pro‐inflammatory, and proliferative responses, particularly in pulmonary tissue. In a similar mechanism, it is possible that effects may also upregulate blood pressure elevations. However, with regard to the tri‐junction of RAAS activation, renal impairment, and hypertension in COVID‐19 infection, the relationship is still unclear. In a large US study of 5700 hospitalized adult patients, the overall rate of hypertension was 56% with similar rates reported in China and Italy (50% and 49%, respectively), identifying overrepresentation of hypertension in severe COVID‐19 cases. It is unclear whether this link represents a causal relationship or confounded by age and other comorbidities [5].

There have been no case reports in pediatrics or adults regarding long‐term renovascular complications following COVID‐19 infection, despite our wealth of knowledge regarding prolonged post‐COVID‐19 complications. Most common sequelae of COVID‐19 infection are characterized by fatigue, dyspnea, headaches, myalgias, olfactory and gustatory dysfunction, and persistent psychiatric symptoms [6]. These symptoms have been documented to persist for even months after initial infection. In a retrospective, community‐based study of multiorgan impairment in low‐risk individuals with post‐COVID‐19 syndrome, patients were assessed with a comprehensive symptom questionnaire, bloodwork, and non‐contrast‐enhanced multiorgan MRI showing inflammation in the kidney in 4% of patients by quantitative T1 relaxation mapping. This study did not obtain patient vital signs or establish the prevalence of hypertension pre‐ and post‐COVID‐19 infection [7]. Clearly, long‐term studies regarding health consequences following COVID‐19 infection, regardless of severity, are necessary to fully understand the spectrum of outcomes in both pediatric and adult patients.

In the pediatric population, current knowledge regarding post‐COVID‐19 infection outcomes is limited given the high prevalence of asymptomatic or mild infection and the difficulty establishing causal association in many studies looking at post‐COVID sequelae [8, 9]. In a literature review, there is a case report of a pediatric patient with presumed COVID‐19 infection (nasopharyngeal swab negative) whose clinical course was complicated by sepsis, acute respiratory distress syndrome (ARDS), acute kidney injury (AKI), and persistent hypertension following symptomatic recovery [10].

While temporally, our patient's hypertension was noted following COVID‐19 illness, we must consider the possibility of alternative etiologies for hypertension, such as obesity, prematurity, and perinatal drug exposure. It is well known that childhood obesity and excess weight are risk factors for the development of essential hypertension. Adolescents born preterm with very low birth weight (VLBW) have also been shown to have higher blood pressure and a higher ratio of plasma AngII to Ang1–7 (indicating imbalance of RAAS system in favor of classic axis) relative to term‐born peers. This association is further compounded by obesity. Obesity may increase the risk of hypertension and cardiovascular disease in individuals born with VLBW by augmenting the prematurity‐associated imbalance of RAAS [11] which notably did not include any differences in PRA or aldosterone levels between study groups. These findings cannot be directly linked to this patient who was an obese adolescent survivor of prematurity, however, without a history of VLBW.

In our patient with persistent hypertension with evidence of hyperreninemic hyperaldosteronism following relatively mild COVID‐19 infection and no evidence of renal artery stenosis on MRA, we must consider the possibility of prolonged post‐COVID‐19 RAAS derangements. In addition, normalization of blood pressure in this patient with low‐dose ACE inhibitor further supports this hypothesis. Increased awareness regarding potential adverse pediatric COVID‐19 outcomes is of particular importance given the high incidence of asymptomatic or mild infection and reduced access to vaccination. These are frontiers for future study into the intricate complexities of interplay between COVID‐19 and RAAS activation.

## Author Contributions

**Vivian Shi:** conceptualization, investigation, writing – original draft. **Teresa Trinka:** project administration, writing – review and editing. **Mohammed Faizan:** conceptualization, investigation, supervision, writing – review and editing.

## Ethics Statement

Our institution does not require ethical approval for reporting individual cases or case series. Identifying information, such as names, images, or specific locations, has been anonymized to ensure participant safety and privacy.

## Consent

Written informed consent was obtained from a legally authorized representative for anonymized patient information to be published in this article.

## Conflicts of Interest

The authors declare no conflicts of interest.

## Data Availability

Data sharing is not applicable to this article as no new data were created or analyzed in this study.

## References

[1] A. C. Simões e Silva , K. Lanza , V. A. Palmeira , L. B. Costa , and J. T. Flynn , “2020 Update on the Renin– Angiotensin–Aldosterone System in Pediatric Kidney Disease and Its Interactions With Coronavirus,” Pediatric Nephrology 36, no. 6 (2021): 1407–1426, 10.1007/s00467-020-04759-1. 32995920 PMC7524035

[2] K. Lanza , L. G. Perez , L. B. Costa , et al., “Covid‐19: The Renin‐Angiotensin System Imbalance Hypothesis,” Clinical Science 134, no. 11 (2020): 1259–1264, 10.1042/cs20200492. 32507883 PMC7276636

[3] American Academy of Pediatrics CsHA , “Children and COVID‐19: State Data Report 2023,” (2023), https://www.aap.org/en/pages/2019‐novel‐coronavirus‐covid‐19‐infections/children‐and‐covid‐19‐state‐level‐data‐report/.

[4] S. Bunyavanich , A. Do , and A. Vicencio , “Nasal Gene Expression of Angiotensin‐Converting Enzyme 2 in Children and Adults,” Journal of the American Medical Association 323, no. 23 (2020): 2427–2429, 10.1001/jama.2020.8707. 32432657 PMC7240631

[5] A. Kanwal , A. Kulkarni , L. Martin , E. Handberg , and E. Yang , “COVID‐19 and Hypertension: What We Know and Don't Know,” (2020), https://www.acc.org/latest‐in‐cardiology/articles/2020/07/06/08/15/covid‐19‐and‐hypertension.

[6] A. Pavli , M. Theodoridou , and H. C. Maltezou , “Post‐COVID Syndrome: Incidence, Clinical Spectrum, and Challenges for Primary Healthcare Professionals,” Archives of Medical Research 52, no. 6 (2021): 575–581, 10.1016/j.arcmed.2021.03.010. 33962805 PMC8093949

[7] A. Dennis , M. Wamil , J. Alberts , et al., “Multiorgan Impairment in Low‐Risk Individuals With Post‐ COVID‐19 Syndrome: A Prospective, Community‐Based Study,” BMJ Open 11, no. 3 (2021): e048391, 10.1136/bmjopen-2020-048391. PMC8727683 33785495

[8] A. Couture , B. C. Lyons , M. L. Mehrotra , et al., “Severe Acute Respiratory Syndrome Coronavirus 2 Seroprevalence and Reported Coronavirus Disease 2019 Cases in US Children, August 2020–May 2021,” Open Forum Infectious Diseases 9, no. 3 (2022): ofac044, 10.1093/ofid/ofac044. 35198651 PMC8860150

[9] P. Kumar and K. R. Jat , “Post‐COVID‐19 Sequelae in Children,” Indian Journal of Pediatrics 90, no. 6 (2023): 605–611, 10.1007/s12098-023-04473-4. 36884145 PMC9992903

[10] S. Bhatta , A. Sayed , R. K. Bhatta , and Y. Acharya , “Rapid Clinical Deterioration With Sepsis and Persistent Hypertension in a Pediatric Patient During Recent COVID‐19 Crisis,” Cureus 13, no. 4 (2021): e14281, 10.7759/cureus.14281. 33959459 PMC8092993

[11] A. M. South , P. A. Nixon , M. C. Chappell , et al., “Obesity Is Associated With Higher Blood Pressure and Higher Levels of Angiotensin II but Lower Angiotensin‐(1–7) in Adolescents Born Preterm,” Journal of Pediatrics 205 (2019): 55–60.e1, 10.1016/j.jpeds.2018.09.058. 30404738 PMC6561332

